# Automating cell counting in fluorescent microscopy through deep learning with c-ResUnet

**DOI:** 10.1038/s41598-021-01929-5

**Published:** 2021-11-25

**Authors:** Roberto Morelli, Luca Clissa, Roberto Amici, Matteo Cerri, Timna Hitrec, Marco Luppi, Lorenzo Rinaldi, Fabio Squarcio, Antonio Zoccoli

**Affiliations:** 1 National Institute for Nuclear Physics, Bologna, Italy; 2 Department of Physics and Astronomy, University of Bologna, Bologna, Italy; 3 Department of Biomedical and Neuromotor Sciences, University of Bologna, Bologna, Italy

**Keywords:** Neuroscience, Image processing, Machine learning, Scientific data

## Abstract

Counting cells in fluorescent microscopy is a tedious, time-consuming task that researchers have to accomplish to assess the effects of different experimental conditions on biological structures of interest. Although such objects are generally easy to identify, the process of manually annotating cells is sometimes subject to fatigue errors and suffers from arbitrariness due to the operator’s interpretation of the borderline cases. We propose a Deep Learning approach that exploits a fully-convolutional network in a binary segmentation fashion to localize the objects of interest. Counts are then retrieved as the number of detected items. Specifically, we introduce a Unet-like architecture, cell ResUnet (**c-ResUnet**), and compare its performance against 3 similar architectures. In addition, we evaluate through ablation studies the impact of two design choices, *(i) artifacts oversampling* and *(ii) weight maps* that penalize the errors on cells boundaries increasingly with overcrowding. In summary, the c-ResUnet outperforms the competitors with respect to both detection and counting metrics (respectively, $F_1$ score = 0.81 and MAE = 3.09). Also, the introduction of weight maps contribute to enhance performances, especially in presence of clumping cells, artifacts and confounding biological structures. Posterior qualitative assessment by domain experts corroborates previous results, suggesting human-level performance inasmuch even erroneous predictions seem to fall within the limits of operator interpretation. Finally, we release the pre-trained model and the annotated dataset to foster research in this and related fields.

## Introduction

Deep Learning models, and in particular Convolutional Neural Networks (CNNs)^1,2^, have shown the ability to outperform the state-of-the-art in many computer vision applications in the past decade. Successful examples range from classification and detection of basically any kind of objects^3,4^ to generative models for image reconstruction^5^ and super-resolution^6^. Thus, researchers from both academy and industry have started to explore adopting these techniques in fields such as medical imaging and bioinformatics, where the potential impact is vast. For instance, CNNs have been employed for identification and localization of tumours^7–10^, as well as detection of other structures like lung nodules^11–13^, skin and breast cancer, diabetic foot^14^, colon-rectal polyps^15^ and more, showing great potential in detecting and classifying biological features^16–18^.

In the wake of this line of applied research, our work tackles the problem of counting cells into fluorescent microscopy pictures. Counting objects in digital images is a common task for many real-world applications^19–22^ and different approaches have been explored to automate it^9,10,23–25^. In the field of natural sciences, many experiments rely on counting biological structures of interest to assess the efficacy of a treatment or the response of an organism to given environmental conditions^26–28^. For example, Hitrec et al.^26^ investigated the brain areas of mice that mediate the entrance into torpor, showing evidence of which networks of neurons are associated with this process. Knowing and controlling the mechanisms that rule the onset of lethargy may have a significant impact when coming to applications to humans. Artificially inducing hibernation may be crucial for a wide variety of medical purposes, from intensive care to oncology, as well as space travels and more. As a consequence, their work arouses considerable interest in the topic and lays the foundations for further in-depth studies.

However, the technical complexity and the manual burden of these analyses often hampers fast developments in the field. Indeed, these experiments typically resort heavily to semi-automatic techniques that involve multiple steps to acquire and process images correctly. In fact, manual operations like area selection, white balance, calibration and color correction are fundamental in order to identify neurons of interest successfully^29–31^. As a result, this process may be very time-consuming depending on the number of available images. Also, the task becomes tedious when the objects appear in large quantities, thus leading to errors due to fatigue of the operators. Finally, a further challenge is that sometimes structures of interest and picture background may look quite similar, making them hardly distinguishable. When that is the case, counts become arguable and subjective due to the interpretation of such borderline cases, leading to an intrinsic arbitrariness.

For these reasons, our work aims at facilitating and speeding up future research in this and similar fields through the adoption of a CNN that counts the objects of interest without human intervention. The advantages of doing so are two-fold. On one side, the benefit in terms of time and human effort saved through the automation of the task is evident. On the other, using a Deep Learning model would impede fatigue errors and introduce a systematic “operator effect”, thus limiting the arbitrariness of borderline cases both within and between experiments.

After outlining a brief overview of related works and stating the contributions of this work, the analysis pipeline is described in the following sections. In “Fluorescent Neuronal Cells dataset”, we describe the data acquisition, the annotation process and peculiar characteristics and challenges of the images. In “Method”, the training pipeline and the experimental settings for the ablation studies are detailed alongside the model architectures compared in our work. In “Results”, the performances achieved by the proposed approaches are evaluated both quantitatively and qualitatively. Finally, “Conclusions” summarizes the main findings of the study .

### Related works

Some interesting approaches have been proposed for detecting and counting cells in microscopic images. In 2009, Faustino et al.^32^ proposed an automated method leveraging the luminance information to generate a graph representation from which counts of cells are retrieved after a careful mining process. Nonetheless, their approach relies on the manual setting of some parameters, like the optimal threshold for separating cell clusters and the luminance histogram binning adopted for retrieving connected components, which hampers the extension to different data.

A few years later, in 2015, Ronnenberg et al.^33^ presented a Deep Learning approach for precise localization (also known as *segmentation*) of cells in an image. Their main contribution is the introduction of a novel network architecture, *U-Net*, which is still state-of-the-art in several applications with only slight adaptations^34,35^. The basic idea is to have an initial contracting branch used to capture relevant features, and a symmetric expanding one that allows for accurate localization. The main drawback is that its enormous number of parameters requires relevant computing power and makes the training difficult because of vanishing gradient^36^. For this reason, a commonly used variation adopts residual units^37^ with short-range skip-connections and batch normalization to prevent that problem. Also, this typically guarantees comparable performance with much less parameters.

A common downside of these approaches is the need of ground-truth labels (or masks) with accurate annotations of whether each pixel belongs to a cell or the background, resulting in an additional and laborious data preparation phase. In an attempt to overcome this limitation, some works tried to tackle the problem in an unsupervised fashion. For example, in 2019 Riccio et al.^38^ addressed segmentation and counting with a step-wise procedure. The whole image is first split into square patches, and a combination of gray level clustering followed by adaptive thresholding is adopted for foreground/background separation. Individual cells are then labeled by detecting their centers and applying a region growing process. While this procedure bypasses the need for ground-truth masks, it still requires handcrafted hyperparameters selection that needs to be tuned for new data. For additional examples of segmentation in biological images, please refer to Riccio et al.^38^.

### Contribution

Our work builds upon Morelli et al.^39^ and it focuses on a supervised learning approach for counting cells (in particular neurons) in microscopic fluorescence, also justifying the output number through a segmentation map that localizes the detected objects. This additional information is particularly relevant to corroborate the results with a clear, visual evidence of which cells contribute to the final counts. The main contributions of our work are the following. First, we develop an automatic approach for counting neuronal cells by comparing two families of network architectures, the Unet and its variation *ResUnet*, in terms of counting and segmentation performance. Second, we conduct ablation studies to show how using weight maps that penalize errors on cell boundaries promotes accurate segmentation, especially in cluttered areas. Finally, we release the pre-trained model (https://github.com/robomorelli/cell_counting_yellow/tree/master/model_results) and a rich dataset with the corresponding ground-truth labels to foster methodological research in both biological imaging and deep learning communities.

## Fluorescent Neuronal Cells dataset

**Figure 1:**
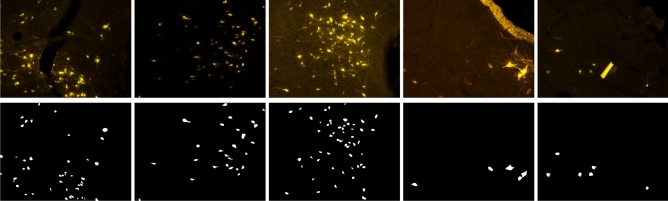
Sample data. The original images (top row) present neuronal cells of different shape, size and saturation over a background of variable brightness and color. The corresponding ground-truth masks used for training (bottom row) depict cells as white pixels over a black background.

The **Fluorescent Neuronal Cells** dataset^40^ consists of 283 high-resolution pictures (1600 × 1200 pixels) of mice brain slices and the corresponding ground-truth labels. The mice were subjected to controlled experimental conditions, and a monosynaptic retrograde tracer (Cholera Toxin b, CTb) was surgically injected into brain structures of interest to highlight only the neurons connected to the injection site^26^. Specimens of brain slices were then observed through a fluorescence microscope configured to select the narrow wavelength of light emitted by a fluorophore (in our case of a yellow/orange color) associated with the tracer. Thus, the resultant images depict neurons of interest as objects of different size and shape appearing as yellow-ish spots of variable brightness and saturation over a composite, generally darker background (Fig. 1, top row).

Although many efforts were made to stabilize the acquisition procedure, the images present several relevant challenges for the detection task. In fact, the variability in brightness and contrast causes some fickleness in the pictures overall appearance. Also, the cells themselves exhibit varying saturation levels due to the natural fluctuation of the fluorescent emission properties. Moreover, the substructures of interest have a fluid nature. This implies that the shape of the stained cells may change significantly, making it even harder to discriminate between them and the background. Combined to that, artifacts, bright biological structures—like neurons’ filaments—and non-marked cells similar to the stained ones handicap the recognition task. Besides complicating the training, all of these factors likewise hinder model evaluation as the interpretation of such borderline cases becomes subjective.

Finally, another source of complexity is the broad shift in the number of target cells from image to image. Indeed, the total counts range from no stained cells to several dozens clumping together. In the former case, the model needs high precision in order to prevent false positives. The latter, instead, requires high recall since considering two or more touching neurons only once produces false negatives.

### Ground-truth labels

Under a supervised learning framework, the training phase leverages ground-truth labels acting as examples of desired outputs that the model should learn to reproduce. In the case of image segmentation, such targets are in the form of binary images (*masks*) where the objects to segment and the background are represented by white and black pixels, respectively (Fig. 1, bottom row).

Obtaining target masks usually requires a great effort in terms of time and human resources, so we resorted to an automatic procedure to speed up the labeling. In particular, we started from a large subset composed by 252 images and applied gaussian blurring to remove noise. The cleaned images were then subjected to a thresholding operation based on automatic histogram shape-based methods. The goal was to obtain a loose selection of the objects that may seem good candidates to be labeled as neuronal cells. After that, acknowledged operators reviewed the results to discard the false positives introduced with the previous procedure, taking care of excluding irrelevant artifacts and misleading biological structures. The remaining images were segmented manually by domain experts. We included significant pictures with peculiar traits—such as artifacts, filaments and crowded objects—in the latter set to have highly reliable masks for the most challenging examples .

Despite the huge popularity Deep Learning has gained in computer vision in the last decade, the lack of annotated data is a common curse when dealing with applications involving non-standard pictures and/or tasks^41^. Since ground-truth labels are expensive to acquire in terms of time and costs, a common approach is to fine-tune models pre-trained on giants datasets of natural images like ImageNet^42^ or COCO^43^, possibly using as few new labels as possible for the task of interest. However, this strategy often does not apply to use cases where the pictures under analysis belong to extraneous domains with respect to the ones used for pre-training^14^. For this reason, by releasing the annotated dataset and our pre-trained model we hope to (i) foster advances in fields like biomedical imaging through the speed up guaranteed by the automation of manual operations, and (ii) promote methodological research on new techniques of data analysis for microscopic fluorescence and similar domains.

## Method

This work tackles the problem of segmenting and counting cells in a *supervised learning* framework. For this purpose, we address the segmentation task exploiting four CNN architectures belonging to the Unet and ResUnet families. Once the cells are detected, the final count is retrieved as the number of connected pixels in the post-processed output. In doing so, we also test the impact of study design choices intended to reduce false negatives and promote accurate segmentation.

### Model architecture

**Figure 2:**
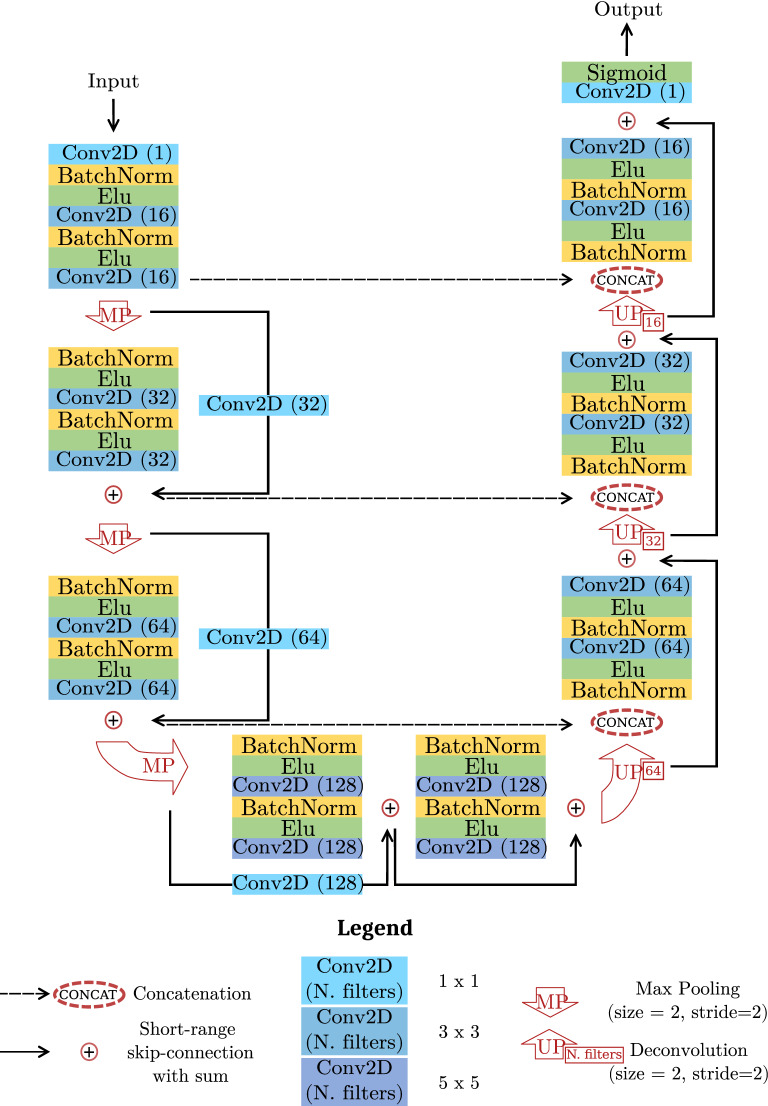
Model scheme. Each box reports an element of the entire architecture (individual description in the legend).

We compare the detection and counting performance of four alternative architectures derived from two network families, Unet and ResUnet, commonly used for segmentation tasks. In the former family, we pick the original Unet architecture^33^ and a smaller version (small Unet) obtained by setting the initial number of filters equal to the ResUnet proposed in Zhang et al.^44^ and scaling the following blocks consequently. In the latter, we pick a ResUnet implementation available in literature^44^ and a similar version with minor modifications. Specifically, we add an initial 1 × 1 convolution to simulate an RGB to grayscale conversion which is learned during training. Moreover, we insert an additional residual block at the end of the encoding path with 5 × 5 filters (instead of 3 × 3). These adjustments should provide the model with a larger field of view, thus fostering a better comprehension of the context surrounding the pixel to classify. This kind of information can be beneficial, for example, when cells clump together and pixels on their boundaries have to be segmented. Likewise, the analysis of some background structures (Fig. 1, top-left image) can be improved by looking at a broader context. The resulting architecture is reported in Fig. 2 and it will be referred to as **cell ResUnet (c-ResUnet)** in the following.

### Ablation studies

Alongside the four network architectures, we also tested the effect of two design choices intended to mitigate errors on challenging images containing artifacts and cells overcrowding.

#### Artifacts oversampling (AO)

The presence of biological structures or artifacts like those in Fig. 1 (rightmost pictures) can often fool the model into detecting false positives. Indeed, their similarity with cells in terms of saturation and brightness, added to the fact that they are underrepresented in the data, make it difficult for the model to handle them correctly. For this reason, we tried to increase the augmentation factor for these inputs to facilitate the learning process. Specifically, we selected 6 different crops representing such relevant structures and re-sampled them with the augmentation pipeline described in *Model training*, resulting in 150 new images for each crop.

#### Weight maps (WM)

**Figure 3:**
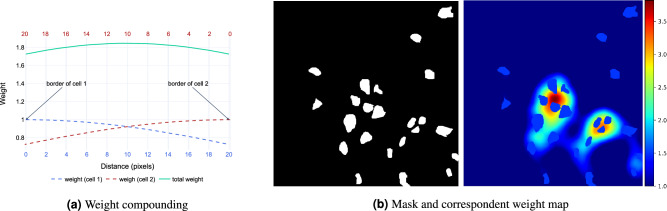
Weight map. 3a shows the weight factors of background pixels between cells according to Eq. (1). The dashed curves depict the weights generated by single cells as a function of the distance from their borders. The green line illustrates the final weight obtained by adding individual contributions. In 3b, a target mask and the corresponding weight map.


One of the toughest challenges during the inference is related to cell overcrowding. As a matter of fact, failing to precisely segment cells boundaries may lead to spurious connections between objects that are separated. Consequently, multiple objects are considered as a single one and the model performance deteriorates. In order to improve cell separation, Ronneberger et al.^33^ suggested leveraging a weight map that penalizes more the errors on the borders of touching cells. Building on that, we introduce a novel implementation where single object contributions are compounded additively. This procedure generates weights that decrease as we move away from the borders of each cell. At the same time, the contributions coming from single items are combined so that the global weight map presents higher values where more cells are close together (see Fig. 3a). The pseudocode for a weight map is reported in Alg. 1, and an example weight map is shown in Fig. 3b.

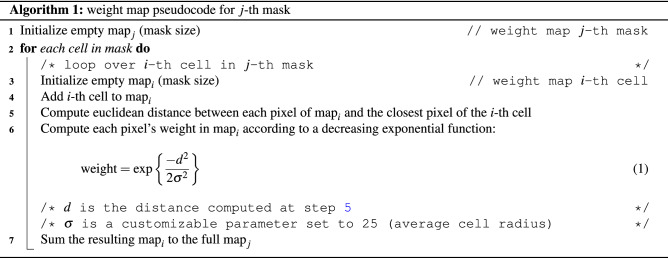


### Model training

After randomly setting 70 full-size images apart as a test set, the remaining pictures were randomly split into training and validation sets. In particular, twelve 512x512 partially overlapping crops were extracted from each image and fed as input to the network after undergoing a standard augmentation pipeline. Common transformations were considered as rotations, addition of Gaussian noise, brightness variation and elastic transformations^45^. The augmentation factors for crops not included in the artifact oversampling ablation study were set to 10 for manually segmented images and 4 to all the others. As a result, the model was trained on a total of nearly 16000 images (70% for training and 30% for validation).

All competing architectures were trained from scratch under the same conditions to favour a fair comparison. Specifically, the Adam^46^ optimizer was employed with an initial learning rate of 0.006 and a scheduled decrease of 30% if the validation loss did not improve for four consecutive epochs. A *weighted binary cross-entropy* loss was adopted on top of the weight maps to handle the unbalance of the two classes (weights equal to 1 and 1.5 for cells and background, respectively). All models were trained until no improvement was observed for 20 consecutive epochs. In this way, each model was allowed to converge and the comparison was made at the best of each architecture’s individual capabilities.

The approach was implemented through Keras API^47^ using TensorFlow^48^ as backend . The training was performed on 4 V100 GPUs provided by the *Centro Nazionale Analisi Fotogrammi* (CNAF) computing center of the *National Institute for Nuclear Physics* in Bologna.

### Post-processing

**Figure 4:**
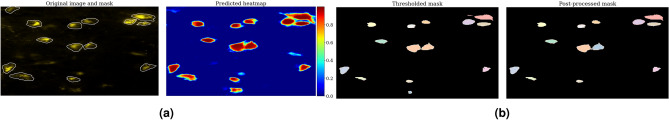
Model output. From left to right, the input image with white contours indicating annotated cells; the model’s raw output (heatmap); the predicted mask after thresholding at 0.875; the predicted mask after post-processing.

The final output of the model is a probability map (or heatmap), in which each pixel value represents the probability of belonging to a cell. Figure 4a reports an example of an input image (left) and the corresponding heatmap (right). The higher the value, the higher is the confidence in classifying that pixel as belonging to a cell. A thresholding operation was then applied on the heatmap to obtain a binary mask where groups of white connected pixels represent the detected cells. Figure 4b (left) illustrates the cells detected after the binarization with different colors. After that, ad-hoc post-processing was applied to remove isolated components of few pixels and fill the holes inside the detected cells. Finally, the watershed algorithm^49^ was employed with parameters set based on the average cell size. An example of the results is provided in Fig. 4b, where the overlapping cells in the middle present in the binary mask (left) are correctly splitted after post-processing (right). Also, the small object in the top-right corner is removed.

### Model evaluation

The Unet, small Unet, ResUnet and c-ResUnet architectures were evaluated and compared based on both detection and counting performance. Also, ablation studies assessed the impact of artifacts oversampling and weight maps.

In order to evaluate the detection ability of the models, a dedicated algorithm was developed. Specifically, each target cell was compared to all objects in the corresponding predicted mask and uniquely associated with the closest one. If the distance between their centroids was less than a fixed threshold (50 pixels, i.e. average cell diameter), the predicted element was considered a true positive (TP); a false negative otherwise (FN). Detected items not associated with any target were considered as false positives (FP) instead. Starting from these values, we referred to accuracy, precision, recall and $F_1$ score as indicators of detection performance. The definitions of such metrics are reported below:

2 $$\begin{aligned} \text {accuracy}&= \frac{\text {TP}}{\text {TP} + \text {FP} + \text {FN}} = \frac{\text {1}}{\text {1} + \frac{1}{\text {TP}} \left( \text {FP} + \text {FN}\right) } ; \end{aligned}$$

3 $$\begin{aligned} \text {precision}&= \frac{\text {TP}}{\text {TP} + \text {FP}}; \end{aligned}$$

4 $$\begin{aligned} \text {recall}&= \frac{\text {TP}}{\text {TP} + \text {FN}}; \end{aligned}$$

5 $$\begin{aligned} F_1 \text {score}&= \frac{2 * \text {precision} * \text {recall}}{\text {precision} + \text {recall}} = \frac{2*\text {TP}}{2*\text {TP} + \text {FP} + \text {FN}} = \frac{\text {1}}{\text {1} + \frac{1}{\text {2TP}} \left( \text {FP} + \text {FN}\right) } . \end{aligned}$$

Notice that we do not have true negatives in Eq. (2) since the prediction of the class “not cell” is done at the pixel level and not at the object level, so there are no “non-cell” objects predicted by the model.

Regarding the counting task, the Mean Absolute Error (MAE), Median Absolute Error (MedAE) and Mean Percentage Error (MPE) were used instead. More precisely, let $n_{\text {pred}}$ be the number of detected cells in *i*-th image and $n_{\text {true}}$ be the actual one. Then, the absolute error (AE) and the percentage error (PE) were defined as:

6 $$\begin{aligned} \text {AE}&= |n_{\text {true}} - n_{\text {pred}}|; \end{aligned}$$

7 $$\begin{aligned} \text {PE}&= \frac{ n_{\text {true}} - n_{\text {pred}}}{n_{\text {true}} }. \end{aligned}$$

Hence, the above counting metrics are just the mean and the median of the AE and the PE.

#### Threshold optimization

The choice of the optimal cutoff for binarization was made based on the $F_1$ score computed on full-size images. In practice, each model was evaluated on a grid of values and the best one was selected according to the *Kneedle* method^50^. The resultant threshold was then used to assess performances on the test set. Although the ultimate goal is retrieving the counts, we relied on detection performance to enforce accurate recognition and avoid spurious balancing between false positives and false negatives that are indistinguishable from the counts. Also, full-size images (and not crops) are used to simulate better the model’s performance in a real-world scenario.

**Figure 5:**
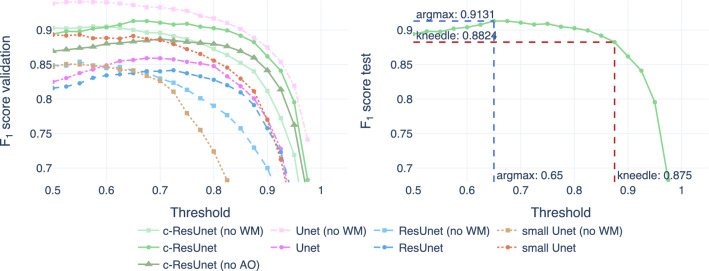
Threshold optimization. On the left, the $F_{1}$ score computed on validation images as a function of the cutoff for thresholding. On the right, the test $F_1$ score of the c-ResUnet model is used to illustrate the selection of the best threshold for binarization according to *argmax* (blue) and *kneedle* (red) methods.

Figure 5 shows the optimization results. On the left, we can see how each model performance varies in the validation set as a function of the cutoff for binarization. Even though lower thresholds work best for all models, the $F_1$ curves are rather flat after their peaks. Thus, increasing the cutoff allows focusing only on predictions whereby the model is very confident, with just a slight loss in overall performance. Also, good practices in natural science applications suggest being conservative with counts and only consider clearly stained cells. For these reasons, we resorted to the *Kneedle* method^50^ for the selection of the optimal threshold. An example of that choice in the case of c-ResUnet is reported in Fig. 5 (right plot).

## Results

After the training, the four competing architectures were compared in three different scenarios: full design, weight maps only (no AO) and artifacts oversampling only (no WM). The 70 full-size images of the test set were used as a testbed. Table 1 reports individual model performances in terms of both detection and counting ability.

**Table 1:** Performance metrics computed on the test set using the optimal *kneed* threshold.

Model	Threshold	$F_1$	Accuracy	Precision	Recall	MAE	MedAE	MPE (%)
**c-ResUnet**	**0.875**	**0.8149**	**0.6877**	0.9081	**0.7391**	**3.0857**	**1.0**	− 5.13
c-ResUnet (no AO)	0.875	0.8047	0.6732	0.9019	0.7264	3.0857	1.5	− 6.24
c-ResUnet (no WM)	0.875	0.7613	0.6147	0.9418	0.6389	3.6857	**1.0**	− 19.14
ResUnet	0.850	0.7855	0.6468	0.8865	0.7052	3.3286	**1.0**	**− 4.84**
ResUnet (no WM)	0.850	0.7513	0.6016	0.9387	0.6262	4.0571	2.0	− 24.12
Unet	0.875	0.7724	0.6291	0.9117	0.6700	3.5143	1.5	− 14.36
Unet (no WM)	0.850	0.7886	0.6510	0.8989	0.7024	3.1571	2.0	− 9.23
Small Unet	0.875	0.7563	0.6081	0.9264	0.6389	3.5714	2.0	− 21.37
Small Unet (no WM)	0.825	0.6697	0.5034	**0.9483**	0.5176	4.7714	2.0	− 32.01

The first four columns report the detection metrics, while the latter ones evaluate counting performance. The best scores for each metric are reported in bold, with underline to highlight the main indicators of interest.

### Performance

By looking at the main figures of merit ($F_1$ score and MAE), c-ResUnet clearly outperforms all competitors. Remarkably, the Unet is consistently worse than c-ResUnet and ResUnet despite having far more parameters (nearly 14M against 1.7M and 887 k, respectively). The advantage of the ResUnet architectures is even more evident when comparing with the lighter Unet version which has a comparable number of parameters (876 k).

In addition, c-ResUnet keeps its leading role also when extending the evaluation to the other metrics. The only meaningful exception is precision, for which the Unet architectures are better. This is probably due to a tendency to “overdetection”. Nonetheless, the ResUnet counterparts well balance this behaviour with a significant improvement in accuracy and recall.

Finally, it is worth noticing that adopting the kneed optimal threshold ensures large cutoffs and enforces only detections with high confidence. Although desired, this behavior also increases false negatives as less cells are detected. As a result, we observe a drop in the accuracy whereby the impact of false negatives is twice as much the one in the $F_1$ score (cfr. Eq. (2) and Eq. (5)), thus explaining the gap between these two metrics. In conclusion, the model provides reliable predictions and satisfies the design requirement of being conservative with counts, as suggested by the negative values of MPE for all experimental conditions.

### Ablation studies

In order to evaluate the impact of artifacts oversampling and weight maps, the experiments were repeated under the same conditions, alternately switching off one of the two design choices.

**Figure 6:**
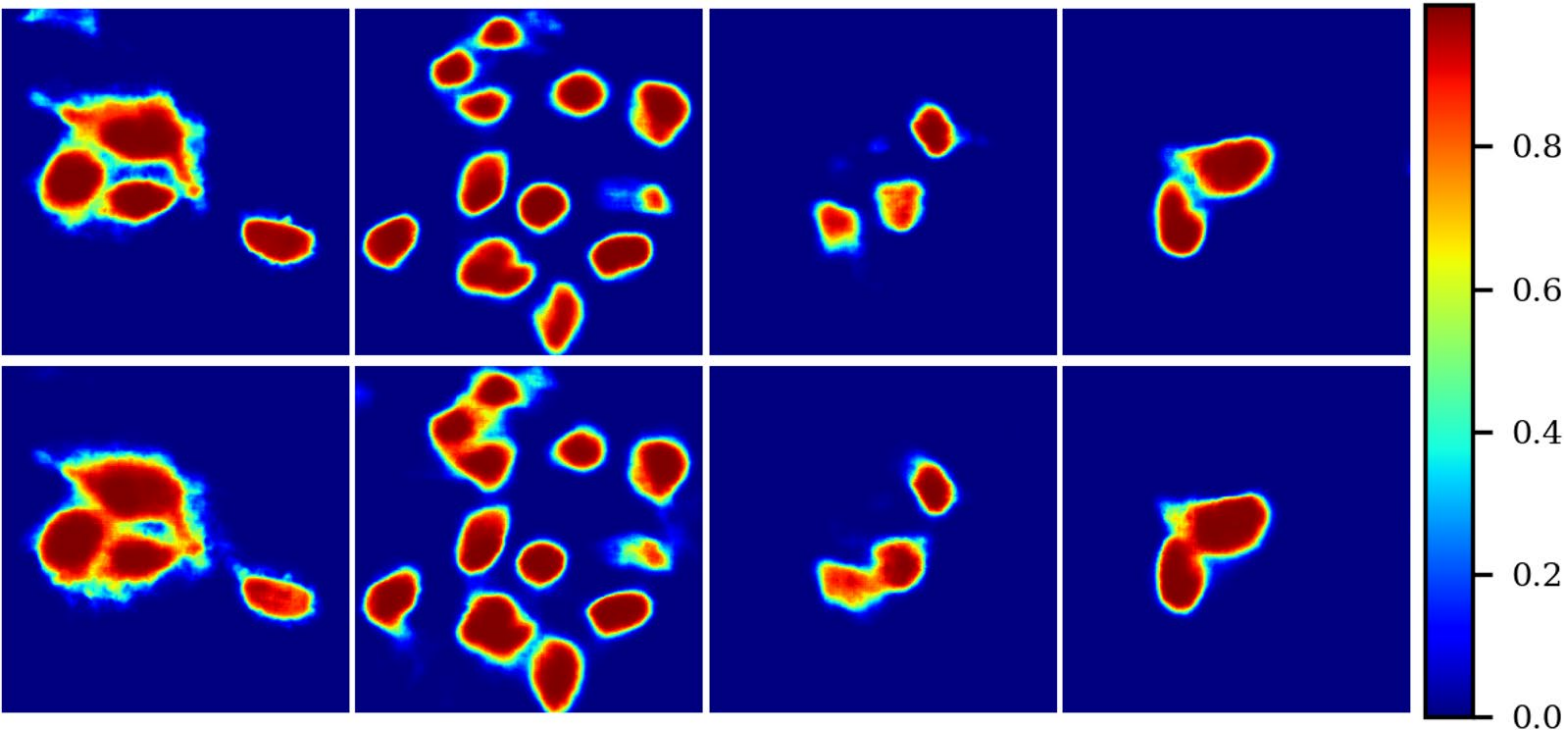
Weight map effect. Predicted heatmaps obtained with c-ResUnet (top row) and c-ResUnet (no WM).

From Table 1 it is evident how penalizing errors in crowded areas has a positive impact. Indeed, experiments exploiting weight maps achieve consistently better results than those without this addition (no WM), except for the Unet architecture. In particular, this strategy seems to produce a loss in precision to foster a more significant gain in accuracy and recall. Fig. 6 illustrates a visual comparison of c-ResUnet output in crowded areas with (top) and without (bottom) weight maps. Again, its beneficial contribution is apparent, with close-by cells sharply separated when exploiting the weight maps.

**Figure 7:**
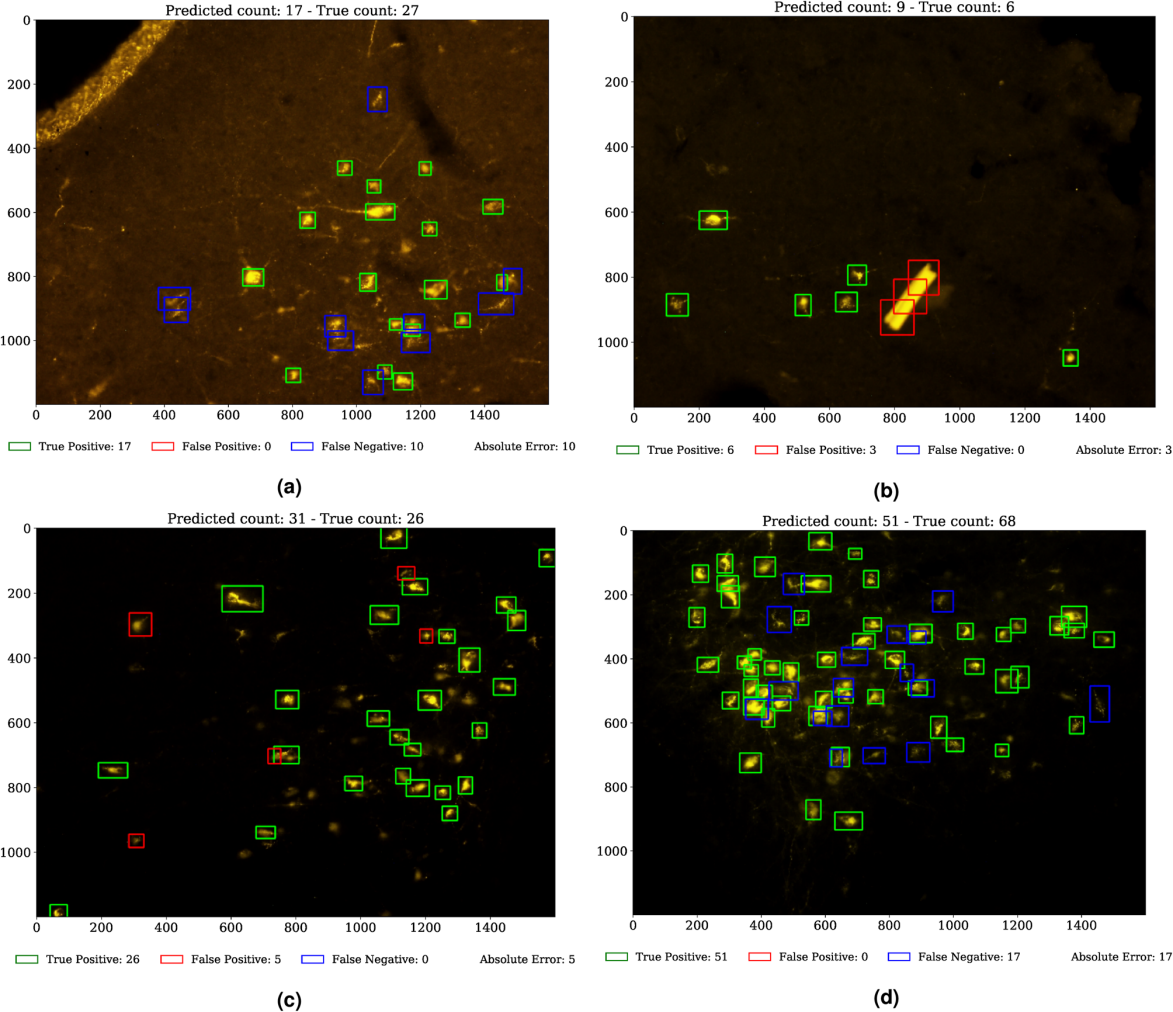
Results on test images. The c-ResUnet (no AO) correctly handles evident artifacts (**a**, topleft corner), while the c-ResUnet fails with more problematic structures (**b**). Notice how false positives (**c**, red boxes) look like target cells. Likewise, the objects discarded (**d**, blue boxes) are similar to other stains that were not annotated.

Regarding the impact of artifacts augmentation, Table 1 shows how there is little difference between the full c-ResUnet and the one without oversampling of challenging examples (no AO). In particular, the advantage of artifacts oversampling is numerically minimal. This is also confirmed by qualitative evaluation (Fig. 7). On the one hand, the c-ResUnet (no AO) is able to avoid detecting more evident artifacts as the strip (7a) even without specific oversampling. On the other, the c-ResUnet still fails to ignore more troublesome bright structures (7b) although additional challenging examples were provided during training. For this reason, the experiment was not replicated for the other architectures.

## Conclusions

In this work, we tackled the issue of automating counting cells in fluorescent microscopy images through the adoption of Deep Learning techniques.

From the comparison of four alternative CNN architectures, the cell ResUnet (c-ResUnet) emerges as the best model amongst the investigated competitors. Remarkably, the careful additions with respect to the ResUnet^44^—i.e. a learned colorspace transformation and a residual block with 5 × 5 filters—enable the model to perform better than the original Unet^33^ despite having seven times fewer parameters.

Also, the two design choices considered in the ablation studies provide an additional boost in model performance. On one side, the adoption of a weight map that penalizes errors on cell boundaries and crowded areas is definitely helpful to promote accurate segmentation and dividing close-by objects. On the other, the effect of artifacts oversampling is less evident. Nonetheless, the combined impact of the two components guarantees better results than any of the two considered separately.

In terms of overall performance, the results are satisfactory. Indeed, the model predicts very accurate counts (MAE = 3.0857) and satisfies the conservative counting requirement, as testified by the negative MPE (-0.0513). Detection performance is also very good ($F_1$ score = 0.8149), certifying that the precise counts come from accurate object detection rather than a balancing effect between false positives and false negatives.

Finally, qualitative assessment by domain experts corroborates further the previous statements. Indeed, by visually inspecting the predictions is possible to appreciate how even erroneous detections are somewhat arguable and lay within the subtle limits of subjective interpretability of borderline cases (see Fig. 7c, 7d).

In conclusion, the proposed approach proved to be a solid candidate for automating current operations in many use cases related to life science research. Thus, this strategy may bring crucial advantages in terms of speeding up studies and reducing operator bias both within and between experiments. For this reason, by releasing the c-ResUnet model and the annotated data, we hope to foster applications in microscopic fluorescence and similar fields, alongside innovative research in Deep Learning methods.

## Data Availability

The original images and the corresponding ground-truth masks are available on AMS Acta, the Open Science repository of the University of Bologna (DOI: http://doi.org/10.6092/unibo/amsacta/6706).

## Code availability

The code is available on GitHub at the link: https://github.com/robomorelli/cell_counting_yellow.
